# Intercept evidence from foreign language communications: Reliability and minimum standards in the interests of justice

**DOI:** 10.1177/13657127241283662

**Published:** 2024-09-19

**Authors:** Nadja Capus, David Gilbert

**Affiliations:** 30777 University of Neuchâtel, Neuchâtel, Switzerland; 195125 Bond University, Robina, Australia

**Keywords:** communication surveillance, fair trial, forensic linguistics, intercept evidence, interpretation, transcription, translation

## Abstract

This paper explores the role of Intercept Interpreters/Translators (IITs) in law enforcement communication surveillance efforts. It focuses on the production and reliability of Translated Intercept Records (TIR), which are comprehensive written records in the target language that may be produced for intelligence purposes or for use in court as Translated Intercept Evidence (TIE). The paper underscores the critical importance of reliable TIR for both evidentiary use and operational decision-making. The authors emphasise the need to establish minimal standards in the quest for reliability and dispel the misconception that literal translations fulfil evidentiary requirements. The standards proposed in this paper aim to minimise interpretation errors to enhance the overall effectiveness of investigations and safeguard the interests of justice. The paper concludes by highlighting the need to align judicial expectations with sound translation practices.

## Introduction

Law enforcement authorities commonly use communication surveillance as an intrusive means of collecting information during their investigations to prosecute criminal activity. Lawfully intercepted communications between drug dealer and client, between bribery demander and supplier, and among members of criminal organisations are a particularly useful source of information for police investigations. Covertly intercepted communications may inform law enforcement authorities about the roles, activities, motivations and plans of people involved in criminal activity. Furthermore, communication surveillance is not only an effective means of covertly collecting information, but it also enables police officers to investigate from a distance without placing themselves in harm's way.

Selected parts of intercepted written communications from various instant messenger services (e.g., Threema, PlayStation, EncroChat, Sky ECC, ANOM, etc.) or from oral intercepted communications obtained from covertly placed listening devices are extensively used as evidence in every EU and Common Law country, including Australia, Canada, France, Germany, New Zealand, Israel and the US. When law enforcement officials and the courts cannot understand the source language of a communication, a suitably qualified interpreter or translator must undertake the task of rendering it into a language that can be understood. There is a terminological muddle regarding how researchers refer to interpreters and translators providing this service (Capus and Havelka, 2022: 1821). In this paper, we propose the term Intercept Interpreter/Translator (IIT). An IIT may be a permanent staff member of a law enforcement agency, or a person engaged under contract with a law enforcement agency.

In many countries, IITs contribute to criminal investigations by producing what we refer to asa *translatum* (*translata* in the plural form, cf. Reiss and Vermeer, 2014: 140), a generic term for all products of interlingual transfer (Griebel and Havelka, 2024: 1). We propose universal application of the term *translatum* to disambiguate references to IIT output. In this sense, the authors propose that IIT output consisting of a verbatim written document in the source language, derived from the intralingual transfer of the intercepted source language in oral form, may be referred to as a transcript, whereas a written document in the target language, derived from the direct interlingual transfer of the intercepted source language in oral form, is more appropriately termed a *translatum*. Both the transcript and the *translatum* are forms of *translata*.

The two primary functions of these *translata* are to serve operational and evidentiary purposes. In an operational context, verbal and written summaries may be produced to provide time-sensitive reporting of relevant information to operational decision-makers. Translated Intercept Records (TIR) are comprehensive written records in the target language which may be produced for intelligence purposes or for use in court as Translated Intercept Evidence (TIE).

As such, *translata* may vary in form from one procedural stage to another and be shared across jurisdictions. For example, *translata* may be transferred via international mutual legal assistance programmes or exchanged within Joint Investigation Teams of Eurojust and the European Union Agency of Criminal Justice Corporation. Joint investigations have been particularly important in recent years due to increased use of encrypted communications by criminal networks (Eurojust, 2023). The tasks that IITs perform for operational and evidentiary purposes fall within the field of criminal law, interpreting (the interlingual transfer from oral to oral and oral to written) and translation (the interlingual transfer from written text to written text) studies, and the broader field of forensic linguistics (Eades et al., 2023).

This paper addresses the questions of what minimal standards are required to realise a satisfactory level of reliability of *translata* produced by IIT and how courts may assess reliability. Generally, prosecutors rely on the assumption that TIR are accurate prior to being admitted as TIE, when in fact serious errors of translation contained in TIE may remain undetected throughout the trial process (Gilbert, 2014: 138). In contrast to monolingual intercepted communications, the reliability of TIE cannot be easily assessed by the trier of fact when the audio recording of the intercepted conversations is in a language other than that normally used in the court. Hence, law enforcement agencies need to be able to provide reliable TIE to the trier of fact, and the trier of fact needs to be able to assess the reliability of the TIE. We define reliability of TIE here as the extent to which the integrity of the primary evidence is preserved.

TIE reliability is explained in detail under ‘Reliability of TIE’. Courts frequently refer to notions of translation accuracy based on the premise that the translation from intercepted communications is a process of word-for-word transfer, including syntactic structure, from one language to another. When the audio recording of an intercepted communication is accompanied by a *translatum*, there is a commonly held misconception among judges and jury members that word-for-word translations are possible to ensure integrity of the evidence (Capus and Griebel, 2021: 92; Gilbert, 2014: 261; Gilbert and Heydon, 2021: 9). Based on current research findings, we consider that this idea of accuracy is not an adequate approach to assess the reliability of TIE noting that the application of context is essential in any translation process (Viaggio, 1992: 32). This is especially relevant when producing TIE to assist the trier of fact to understand the evidence contained in audio recordings of intercepted conversations. Translation approaches that remain close to form, or translate word-for-word, without adequate explanation by the translator in the form of notes where necessary, risk rendering the resultant *translatum* incomprehensible to the trier of fact. The IIT's dilemma is to produce TIE conveying meaning of the source language while also preserving the integrity of the intercepted evidence as required by the court. The IIT may produce a *translatum* that does not convey naturalness in the target language but may be considered by the courts to satisfy evidentiary requirements of accuracy. This is because the TIE appears to mirror the words and syntactic structures of the source language. The tendency of IIT to translate close to form, or more literally, results in the TIE making sense in some sections but not in others. For this reason, the authors use reliability as a core concept to address the quality of TIE by identifying and proposing minimum standards. The minimum standards proposed in this paper offer a basis from which a uniform approach can be applied to evaluate the reliability of the evidence, aligning judicial expectations with translation practices.

As a basis from which to propose minimum standards of producing reliable TIE, the authors first describe in detail how law enforcement authorities engage the IIT to assist with ongoing investigations and the collection of evidence. This is important to understand the complexity of the activity and to shed light on the extent to which evidence is likely to be impacted through the translation process (Capus and Griebel, 2021: 74; Capus and Havelka, 2022: 1821; Gilbert, 2014; Havelka, 2024). Second, the authors elaborate on the concept of reliability considering the specificity of TIE, positing that for TIE to be reliable in a broad sense, evidentiary as well as translational requirements of reliability must be met (Capus et al., 2024). From this basis, the paper proposes minimum standards that should be respected when producing and using TIE to mitigate prospects of interpretation/translation error. Reliability is also crucial when IITs undertake operational interpreting tasks during investigations, as operational effectiveness and the interests of law enforcement officials and suspect(s) are all affected. Erroneous intercept interpreting may result in ineffective decision-making, placing law enforcement agents at elevated risk when engaged in operational activity, such as conducting raids or arrests. Therefore, there is also a risk that the investigation becomes inefficient, and ineffective when relying on ambiguous or misinformation contained in *translatum*. Fundamental rights of wrongfully suspected persons may also be violated. While this paper focuses on reliability of TIE, we underline the necessity of ensuring reliability of any *translata* that IITs produce during operational phases of the investigation. While we propose minimum standards in this paper to improve operational and evidentiary processes, it is not our intention to prescribe how minimum standards should be implemented or operationalised in individual legal systems. The paper ends with some concluding remarks emphasising the need to further align judicial expectations with translation practices.

## IIT—Operational support and evidence gathering

Investigations into suspected or actual criminal activity may depend on time-sensitive information interpreted from live intercepted communications. Police often rely on information from intercepted phone calls or from listening devices to make an arrest, initiate a raid or take action to prevent injury, death or property damage. Law enforcement authorities may also engage the IIT to perform tasks to meet intelligence collection requirements. For example, communication surveillance in the form of telephone interception may be required to collect information about the activities of a particular individual or group of interest suspected of being engaged in criminal activity. In such cases, the IIT will produce summaries relating to targeted criminal activity. IIT may also assist intelligence analysts with breaking codes, establishing patterns of speech, language and behaviour, and/or determining the organisational structure of a particular group.

Activities specific to the IIT role includes monitoring, selecting, translating, deciphering and transcribing the intercepted communication in close cooperation with police officers (Capus and Havelka, 2022: 1830; Drugan, 2020: 318; González Rodríguez, 2015). Hence, the tasks that IITs undertake go well beyond the general practice of interpreting or translating, culminating in a hybrid activity, an activity *sui generis* (Capus and Havelka, 2021: 191; 2022: 1822). IITs face significant challenges due to the nature of intercept interpreting, even when intercepted conversations are clearly heard and expressed in simple language. IITs are not a party to the communication event that they are intercepting but are observing and recording. This is an important point because consistency and context are essential elements of communication considering that the source language speaker has expressed the utterance in a manner that is of optimal relevance to the intended recipient (Gutt, 1998: 51). Therefore, as an external observer, the IIT assumes the contextual understanding shared between participants in the communication event and intervenes where necessary in the translation process based on the IIT's assumption of what is meant by what is said in the source utterance (Gilbert, 2014: 134). This setting is in stark contrast to the tasks undertaken by community or court interpreters/translators, who usually engage in, and facilitate the exchange of, communication between participating actors (González et al., 2012: 967 ss.). Interpreters and translators in community or court settings can ask questions, seek clarification and take body language of the source language speaker into account. In contrast, the IIT may be unaware of the context within which the intercepted conversation takes place. IITs may also be confronted with indistinct speech and extralinguistic sounds, such as background noise, that may make the source language difficult to hear or to understand. In such circumstances, the IIT does not have the opportunity to seek clarification, request repeats or to observe non-verbal cues.

The role of the IIT in support of law enforcement operations is not dissimilar to that of a military cryptologic linguist, where both aim to provide usable information derived from the interpretation/translation of intercepted communications in support of operational objectives (Gilbert, 2014: 7). The point of departure in terms of output is where IITs are tasked with producing *translata* for evidence purposes in the form of TIE. Cryptologic linguists provide translations primarily for operational or intelligence purposes. Another clear distinction in terms of training is that cryptologic linguists are highly trained in communications interception and operational interpreting with the requirement that they have exceptional comprehension and analytic skills. In contrast, IITs are usually interpreters and translators who have not received specific training in the skills required of communications interception and operational interpreting (Gilbert, 2014: 124). Research has established the need for IITs to be trained in the skill sets required to perform the tasks of operational interpreting and producing TIR/TIE, and the requirement for the introduction of standards has been identified (Capus and Bally, 2020; Capus and Havelka, 2022: 1832; Gilbert, 2014: 264; Taibi and Martin, 2012: 79). This deficiency in IIT training is concerning, considering that law enforcement authorities throughout the world increasingly rely upon support from IIT to combat trans-national criminal activity. Criminal activity includes threats to national security, such as terrorism, cyber-attacks, and the illicit-drug trade (Grabosky and Stohl, 2010: 71). It follows that appropriate IIT training should not only be recognised as necessary to uphold the principles of justice but also to effectively contribute to national and global security.

The nature of operational interpreting and translating requires IITs to take all available information into account to provide the decision-maker with time-sensitive information of relevance to the operation/investigation. This may be provided in the form of short verbal or written summaries of what was heard or written in intercepted communications. In such cases, using translation strategies that remain faithful to the source language are not likely to produce a report that is usable in time-sensitive operations. The report needs to be clear, concise, accurate, relevant and, most importantly, reported in an appropriate format to support effective decision-making. Therefore, the appropriate translation strategy applied for time-sensitive operational reporting will usually take the form of a meaning-based approach, having considered all contextual information available to the IIT at the time of making the report. This strategy is necessary to optimise the value of information provided to the decision-maker, considering that the significance of information is influenced by its nature and the context within which it is interpreted (Heuer, 1999: 42).

IITs may also be required to assist law enforcement officials with complex and detailed analysis of intercepted communications. Criminals may use codes and veiled speech to disguise the true nature of their activities (Nunn, 2010: 34). Not unlike the role of a cryptologic linguist in the military, IITs are likely to be called upon to apply their language and analytic skills to crack cyphers and to assist with making sense of intercepted communications containing veiled speech or coded language (Gilbert, 2014: 255). The interpretation of intercepted text messages containing emojis, abbreviations in a mix of languages, and acronyms also presents challenges to IITs (Loiacono, 2022: 92) The methodology that the IIT applies when performing analytic tasks of this nature is dynamic. Translation approaches that remain true to the form of the source language and that apply a more dynamic approach conveying sense in the target language, are both necessary to determine the underlying meaning of coded language (Gilbert, 2014: 231).

Considering the distinction between IIT roles of operational interpreting and that of producing *translata* for evidentiary purposes, it is essential to identify the variance in approaches to the translation process for each purpose. When producing TIE, the IIT is required to produce a document in the language of the court that mirrors what the IIT perceived in the intercepted communication. In contrast to *translata* for operational purposes, TIE is neither perishable nor time-sensitive. Instead, courts generally require the TIE to be complete and not fused with intelligence or background contextual information. For evidentiary purposes, the courts require that TIE represents an accurate transfer of communication from the source into the target language (Gilbert, 2014: 223). This approach towards finding equivalence during the interlingual transfer process aligns with what may be referred to as a formal or more literal approach which seeks to retain, as closely as possible, the linguistic elements of the source text (Nida, 1964: 2). However, this approach has significant limitations when framed within the context of TIE, as Taibi and Martin (2012) show based on the case of Taysir Alony, the Al-Jazeera reporter who was imprisoned because of alleged collaboration with a terrorist organisation. Analysing several examples, Taibi and Martin demonstrate the inappropriateness of excessive literal translation (2012: 81). Literal translations that fail to take pragmatic and cultural aspects into account should be accompanied by translator's notes.

Misinformed notions of accuracy held by the courts create a serious dilemma for the IIT. Courts expect TIE to be narrowly translated and not to reflect the IIT's analytical and contextual perspective that is usually applied when supporting law enforcement operations. Translation studies have long established that interlingual transfer always includes interpretation (Nida, 1964: 156; Viaggio, 1992: 31). Additionally, it is generally accepted that no two languages are the same in terms of lexicon, semantics or syntactic structure; therefore, there can never be a perfectly accurate translation. While formal equivalents may not exist between the source language and the target language, an equivalent effect may be achievable (Nida, 1964: 156). A dynamic approach through translator intervention when producing TIE is likely necessary to convey sense.

Research involving linguistic analysis of TIE has demonstrated that IITs, like interpreters and translators in general, do not act as mere conduits (Angelelli, 2004; Gilbert, 2014; Laster and Taylor, 1995: 10). When the source language is translated word for word, the resultant translation is usually nonsensical in the target language. A translation approach that remains closely aligned to the source language structure and content without conveying sense can produce ambiguity and distortion in the target language. An example of the difference between a transcript of the source language (Vietnamese), a word-for-word translation into the target language (English), the corresponding TIE used in court (English) and a proposed correct translation (English), provided in the example below. The example is of a lawfully intercepted conversation using a listening device placed in a kitchen. Two people were present in the room, and one was dividing compressed heroin with a knife. Each time the person cut the block of heroin, some of it would crumble away. The person dividing the heroin became frustrated, and the proposed correct translation below reflects this. However, by remaining too close to the form of the source language (translating too literally), the IIT failed to convey sense, which resulted in significant confusion in the court room.

The following is an example of TIE containing idiomatic expression (Gilbert, 2014: 94):

**Table:** 

Source language (Vietnamese)	Chia-là-mất,-chết.
Word-for-word translation	Divide-is-lose,-dead.
TIE	Lose it, God oh God, is it dead?
Proposed correct translation	Dividing (it) means losing (some), damn it!

Providing a word-for-word translation (line 2) of the source language transcript (line 1) demonstrates what one may expect of the IIT acting purely as a conduit. Using the example above, we can see that the translator has intervened in the translation process to render the language appearing in the TIE (line 3). However, in this example, the TIE is considered unreliable as is evidenced by the proposed correct translation (line 4). This example demonstrates how a narrow approach to translation can disadvantage both the prosecution and the defence, as the IIT has remained too close to the form of the source language, translating it in a rather awkward way. The literal meaning of the word ‘chết’ in Vietnamese is ‘dead’, as it appears in the TIE. In the context of the source utterance, the word ‘chết’ is an idiomatic term expressing the emotion of frustration as seen in the proposed correct translation where the word ‘chết’ is translated as ‘damn it!’ It became evident during the trial that even the prosecution was confused about who or what was dead.

During questioning, the prosecution asked the defendant, giving evidence in their defence, what they meant when they said in Vietnamese, ‘Lose it, God oh God, is it dead?’ The prosecutor rephrased the question and asked, ‘Who or what was dead?’ The Vietnamese court interpreter interpreted the question posed by the prosecutor with no scope to convey the idiomatic meaning of ‘chết’ as it appeared in the original utterance. The defendant was confused, and the prosecutor became frustrated. The court interpreter knew what the problem was but remained impartial for ethical reasons. The witness was unable to respond in a way that appeased the prosecutor because the idiomatic meaning was lost in the TIE, and therefore lost in the court interpreting process. Confusion continued in the courtroom until the issue was resolved with the assistance of another court interpreter present (Gilbert and Heydon, 2021: 7). The above example demonstrates the importance of IITs conveying sense wherever possible and providing translator's comments where necessary.

Previous research has shown that the IIT shifts between producing a translation remaining as close to the form of the original language as possible (often described as a literal approach), and a more flexible and creative approach that focuses on conveying sense in the target language (a free approach). The difference between these approaches is that the approach remaining close to the form of the target language often requires the intended readership to have some understanding of the linguistic and cultural context of the source language, in contrast to the dynamic or creative approach, which aims to produce a seamless translation that is linguistically and culturally adapted to the target readership. It is often necessary for a formal approach to be compromised to convey sense in the target language, in which case translators will need to provide notes or comments for the translation to make sense (Nida, 1964: 159). This is particularly relevant when the culture associated with the source language is vastly different from that of the target language (Nida, 1964: 161). The fluctuation between formal and dynamic translation approaches often results in incoherent sections of TIE. This is particularly prevalent when IITs translate word-for-word because they are in doubt as to what the intercepted words mean when context is unclear or unknown. Incoherent shifts in language style appearing in TIE make it difficult for the trier of fact to understand what is alleged to have been said and what was meant in the source language. Mistranslations contained in TIE may be attributed to IITs not having access to contextual information of relevance, resulting in word-for-word translations becoming more frequent. Frequent shifts between remaining true to the form of the source language and applying dynamic and creative approaches to convey meaning in the target language often result in awkward and disjointed language appearing in TIE. Such TIE may be challenged by the defence citing claims of unreliability due to the TIE being inaccurate or incorrect.

## Reliability of TIE

TIE helps the trier of fact understand the source language (a language other than that of the court) contained in audio recordings and text obtained from telephone intercepts, covert listening devices and other sources (González et al., 2012: 965 ss). Intercepted audio recordings are generally referred to as primary evidence, and TIE is referred to as secondary evidence produced to assist the trier of fact (Fishman, 2006: 487). How the IIT approaches the task of producing TIE is a significant factor that will influence the trier of fact when assessing weight given to TIE. Research has shown that even monolingual transcripts of poor-quality audio recordings risk priming the perception of the trier of fact (Fraser et al., 2011; Fraser and Stevenson, 2014). In this context, priming can be described as the effect of a transcript influencing the perception of the trier of fact to align with that of what the transcript purports to say. In contrast to monolingual transcripts, TIE poses even a greater risk that the trier of fact will readily accept the secondary evidence presented in a language they understand, as they are unable to understand the primary evidence in the form of an audio recording in a language other than that of the court. Hence, two main shifts affect reliability when producing TIE: the transference from one language to another, and the shift from oral to written form. Because of these shifts, many factors contained in the primary evidence are inevitably lost, which would otherwise—in a monolingual situation—be perceived and understood by the trier of fact had the primary evidence been recorded in a language of the court (for a detailed analysis of this multi-layered translational process, see Havelka, 2024). Also, considering the large volume of information collected, the IIT will necessarily filter out non-relevant information and produce summaries so that the information reported is more suitable for operational decision-making.

Police officers, prosecutors, defence lawyers, judges and jury members depend on the *translatum* provided by IITs, which underlines the importance that *translatum* reliability criteria are established, mutually understood and implemented by all parties (González et al., 2012: 968 ss). To identify reliability criteria in a holistic context is complex but is essential in the pursuit of a fair trial. The particularity of TIE is derived from a hybrid nature of the translational activity, which is inextricably linked to criminal law procedure. For this reason, reliability criteria of TIE need to consider evidentiary requirements and translational methodologies used to produce TIE.

Due to the heterogenous nature of national evidentiary rules, the process of producing TIE varies across jurisdictions, not only between Civil Law and Common Law countries, but also between countries using the same legal system. To illustrate this variance, a few examples are briefly explored. For example, in the United Kingdom, intercept material stemming from communication surveillance cannot be adduced as evidence (cf. Regulation of Investigatory Powers Act 2000). There are, however, four exceptions: communication surveillance (including interception of phone calls), recordings from the use of listening devices, interceptions derived from other countries and intercepted phone calls made from prisons.

While in both Common Law and Civil Law countries the sounds and tones recorded on an audio file are considered by the court as primary evidence and the TIE as secondary evidence, the rules of evidence differ regarding whether audio records must be played aloud in court so the defendant has an opportunity to hear the primary evidence against them. Rules also differ considerably between jurisdictions regarding the point in time when defendants must be provided the opportunity to learn about the existence and content of the TIE.

Countries’ legal requirements may also vary in relation to the production of secondary evidence. In many countries, such as Australia, Austria, Germany and Switzerland, intercepted communications are directly transferred into the written target language (Capus and Havelka, 2021: 182s.). In contrast, the US has established a two-step process as the preferred method in accordance with guidelines published by the U.S. National Association for Judiciary Interpreters and Translators (NAJIT, 2019: 1). According to NAJIT guidelines, the intercepted oral communications are first transcribed into the written source language in accordance with prescribed conventions of transcription. The second step requires the transcription in the source language to be translated into the target language, usually the language of the court. In some countries, such as the US, the IIT may be called as a witness to establish the methods they used to produce the *translatum* (Fishman, 2006).

Evidence rules are notably different regarding the (in)admissibility of TIE. In Australia, for example, even if there exists the possibility or likelihood that evidence has been falsified, it may still be found to be relevant, and therefore admissible (Heydon, 2015: 105). In jury trials, judges consider the weight of relevant evidence within the context of the case. Relevant evidence under Australia's Uniform Evidence Law is defined as ‘evidence that, if it were accepted, could rationally affect (directly or indirectly) the assessment of the probability of the existence of a fact in issue in the proceeding’ (Evidence Act 1995 [Cth]—Section 55). Therefore, the threshold for admissibility of evidence is low (Heydon, 2015: 103). The rationale is that evidence of the slightest probative value should not be withheld from the jury unless the probative value of the evidence is outweighed by the danger of unfair prejudice to the defendant.

Notwithstanding such differences in relation to the reliability of TIE, the legal threshold required can be directly deduced from the internationally recognised principle of a fair trial. This principle has materialised into the fundamental right of a fair trial in the European Convention on Human Rights (Article 6), the American Convention on Human Rights (Article 8), the African Charter on Human and Peoples’ Rights and the accompanying Proclamation of the African Commission on Human and Peoples’ Rights of the Principles and Guidelines on the Right to a Fair Trial and Legal Assistance in Africa (African Union, 1992) and the International Covenant on Civil and Political Rights (Article 14; Momsen and Willumat, 2024). The principle underpins the criteria that must be met for reliable TIE. This is regardless of jurisdiction-specific evidentiary rules aimed at realising procedural fairness (cf. for the same approach taken in the context of digital evidence: Stoykova, 2023). Procedural fairness is concerned with fairness of the procedure leading to a decision, and not to the fairness of the decision itself (ALRC, 2013). Procedural fairness serves the interests of justice, balancing the interests of the defendant against those of the community. Therefore establishing minimum standards for intercept interpreting protects both the State and the individual based on the premise that the way in which evidence is obtained, and in this case translated, must be fair.

By intersecting fundamental legal requirements that underpin the principle of fair trial with translational requirements, the authors identify the following two reliability criteria for TIE: impartiality and verifiability (Capus et al., 2024).

### Impartiality

The right to an impartial and balanced criminal investigation intersects with the translatory scope to render a reliable *translatum*. IIT choices made while producing a *translatum* are subject to a range of influences including context, professional ability, time constraints, environmental circumstances, workplace biases and other factors that ultimately affect the reliability of TIE.

Research reveals that workplace environments influence the strategies IITs use to produce TIE, which may affect IIT impartiality (Capus and Havelka, 2022: 1830; Nunn, 2010). Additionally, institutional gatekeeping (the discretionary reporting of information) is a known phenomenon in relation to interpreting. Research on hospital interpreters found that interpreters working for large organisations ‘are not, and cannot be, “neutral” machines of linguistic conversion, both because they are faced with the reality that linguistic systems are not ‘the same’ in how they convey information contextually, and also because they are themselves social agents and participants (albeit special ones) in the discourse’ (Davidson, 2000: 401). Interpreters felt empowered to ensure that corporate objectives were met by selectively interpreting parts of conversations that they felt were relevant to keep patients on track. The invisible role of interpreters in such situations allows for errors of interpreting to remain undetected. Importantly, the expectation that interpreters do not act outside their interpreting role presents a risk that, when they do, the practice may not be detected (Davidson, 2000: 401s.). IITs’ impartiality may be similarly affected as they participate in achieving corporate objectives when working with police, prosecutors and the courts. This is a key point since the translatory process is one of interpretation itself. Moreover, IITs have considerable discretion regarding what is, and what is not, reported in TIE (cf. for more information about IITs gaining ‘co-authorship’: Capus and Grisot, 2023: 382, 392). Impartiality is at risk, as IITs are embedded in the investigation process (Capus and Grisot, 2023: 381). Drugan refers to this arrangement as being ‘a complex collaboration’ with the police (Drugan, 2020), notably when filtering and minimisation are undertaken. This in turn leads to what is referred to in the literature as the entextualisation and recontextualisation of communication regarding intralingual transcription (Nunn, 2010: 31; Park and Bucholtz, 2009). Interlingual transfers from oral communication to TIR are, however, subject to the same mechanisms (Capus and Havelka, 2022: 1824). Selected parts of the conversation are entextualised when extracted from the original context (and out of longer conversations) and subsequently recontextualised when put into a TIR. The TIR is then passed from the police to the public prosecutor and eventually used as TIE. During this process, discourse strategies may be applied so that translations are framed to fit in with the objectives of the dominant discourse (Taibi and Martin, 2012: 78). Indeed, according to Bucholtz, ‘a bias in legal transcription in favour of those who hold the greatest institutional power—judges, lawyers, and police officers’ (2009: 507) is a consistent pattern of transforming speech into text in legal settings.

Therefore, notions of impartiality based on the expectation that IITs provide complete and accurate *translata*—confusing literal translation with accurate translation—are opaque and misleading (Taibi and Martin, 2012: 82). Descriptors of accuracy and completeness, without appropriate qualification, are arbitrary concepts that leave TIE susceptible to distortion, omission and unjustified additions, noting that producing a *translatum* is always a decision-making process (Mansell, 2007: 3)

Importantly, the impartial role of IITs is not dissimilar to that of court interpreters, in the sense that they are both required to negotiate the transfer of meaning from one language to another in the interests of justice. IITs, like court interpreters, must perform their duties impartially and effectively to assist the court. However, there are considerable differences between court interpretation and the roles performed by IITs as mentioned above under ‘IIT—Operational support and evidence gathering’. Court interpreters actively participate in courtroom communication to realise mutual understanding by analysing and reproducing utterances within the prevailing context. (Jacobsen, 2004: 248). In contrast, the IIT is an observer to the communication event and does not participate. Even if the IIT does have access to contextual information, research shows that the IIT may not be permitted to consider extralinguistic knowledge during the translation process, as it is likely to skew the TIE away from mirroring the primary evidence. On the other hand, word-for-word translations often do not make sense. Therefore, it is essential to explore how the IIT comes to understand the context of the communication event. Research shows that there is no consistency in the way law enforcement authorities prepare IITs to produce *translatum* from communications surveillance tasks. The data reveals that law enforcement authorities take heterogeneous approaches, sometimes providing IITs with a briefing that includes background information about the intercepted communications (Capus and Havelka, 2022: 1827) and other times providing little or no background information at all (Gilbert, 2014: 125–127).

Denying IITs access to intelligence or background information bears the considerable risk of them making incorrect assumptions of context when producing TIE. If, on the other hand, the IIT does not make explicit what they know about the operation when producing a TIE, potential violations of impartiality and errors in TIE may go undetected. A study conducted in Australia on Vietnamese drug-related cases found that court interpreters were reluctant to alert the court to errors in TIE because of their ethical requirement to remain impartial (Gilbert, 2014: 141). A defence lawyer participating in the research stated that nobody really knows whether the TIE is accurate or not (Gilbert, 2014: 214). We argue that IITs should approach the translational process when producing TIE no differently from how a court interpreter produces *translata* in a courtroom (i.e., in a form that seeks to convey optimal equivalence within the prevailing context of the communication event).

In casefile-based criminal justice systems, such as that of Switzerland, courts have developed the standard that in cases where the IIT has extralinguistic knowledge of a particular investigation, then that knowledge, or context, should be declared as a translator's note for the purposes of transparency (Capus and Griebel, 2021). That way, the trier of fact can fairly assess IITs’ lexical choices that appear to lean towards the context of alleged criminal activity. In countries such as Australia and the US, however, the IIT may only give opinion evidence if called upon as a witness, such as during a voir dire, to clarify or correct segments of TIE. Even then, the IIT must confine their opinions to areas of specialised knowledge gained through training, study or experience. For example, IITs will not usually be permitted to explain the meaning of drug-related jargon or codewords, as they are not normally considered experts in that area. There have been calls for the TIE to be admitted as expert evidence, rather than simply as an aid to the trier of fact. This may allow the IIT to freely apply, and make explicit, contextual extralinguistic information when producing TIE. If the TIE was to be accepted as expert opinion evidence, then the rules of evidence that apply to expert witnesses would also apply to IITs producing TIE (Fishman, 2006). The distinction between TIE being admitted as expert opinion evidence and being admitted as an aid for the trier of fact has a significant impact on how the TIE is produced and what scope the IIT has in relation to expressing an opinion. When TIE is presented as an aid to the jury, the courts continue to accept the notion that no opinion is offered by the IIT, and that the TIE simply mirrors what was contained in the source language stored in an audio file. If one accepts that the translation process is inherently a decision-making process that IITs undertake, then this view dispels the myth that IITs offer no opinion when TIE is presented as a mere aid to the trier of fact. Consequently, Swiss courts do ignore interpretations provided by IITs. For example, when an IIT states in the TIE that the defendant may have referred to drugs when talking about car wheels, judges may conclude for themselves, based on other sources available to the criminal proceeding, that car wheels were in fact drugs, but they do not base their conclusion on the IIT's opinion.

In sum, a judicial approach depriving IITs of the ability to apply all relevant information of which they are aware is counterproductive to ensuring impartiality. This practice bears the risk of masking underlying biases that may go undetected in TIE and allows the prosecution or the defence to further interpret the TIE in creative ways to advance the interests of their respective objectives. Hence, regardless of how courts view and use the TIE, it is essential that IITs are permitted to provide translator's comments where necessary. Therefore, minimum standards that transcend organisational boundaries are essential to mitigate the risk that IITs make translational choices that are biased in favour of the prosecution case. Only minimum standards that allow IITs to annotate and make comment on their choices when producing *translata* will allow verifiability, the second essential element of TIE reliability.

### Verifiability

Verifiability is an essential element of procedural fairness requiring the disclosure, where necessary, of why translatory choices are made.

An example of where transparency was required of an IIT, but not provided, was where the word ‘kilo’ in Spanish was translated incorrectly in TIE. In Spanish, the word ‘kilo’ has two different meanings depending upon the cultural background of the speaker. When used by a Cuban speaker, it usually means ‘cents’, but a Spanish speaker from elsewhere may use and understand the word to mean ‘kilograms’. Ambiguity concerning this word occurred in a drug-related trial in the US where the accused was eventually acquitted once the ambiguity in the TIE was resolved in the accused's favour (Shulman, 1993: 176). In this case, the IIT heard the accused, from Cuba, use the word ‘kilo’ when talking to another Spanish speaker during an intercepted conversation. The trial had already begun when a court interpreter alerted the court to the ambiguity of the word ‘kilo’ as it appeared in TIE. The court held that the word had been mistranslated and the accused was acquitted. We propose that the IIT should have made a translator's comment if, at the time of producing the TIE, they knew that there was an alternative meaning to the word ‘kilo’.

Another example is where the word ‘ấy’ in Vietnamese has proven to cause significant confusion in the courtroom when the IIT has failed to disclose, in TIE, the dynamic meaning potential of the word. Struggling to find an appropriate English equivalent for the word ‘ấy’, the IIT improvised and used the term ‘thingy’. The word ‘thingy’ became so entrenched in Vietnamese drug-related TIE that police officers gave expert evidence to the effect that ‘thingy’ meant drugs, when in fact it is simply used in Vietnamese as an anaphoric or exophoric reference word meaning ‘this’, ‘that’ or ‘it’ (Gilbert, 2014). The practice of Vietnamese IITs using the word ‘thingy’ in drug-related TIE became systemic within law enforcement authorities in at least two Australian states over a period of at least 14 years (Gilbert, 2014: 161). The potential damage the systemic mistranslation may have caused to the interests of justice in previous cases was exposed during a voir dire where the IIT gave evidence relating to the word ‘‘thingy’. When asked by the defence counsel what ‘thingy’ means, the IIT responded:

Sometimes we could not define or know what the meaning of it is so we use that word, just for something or someone or could be whatever. (Gilbert, 2014: 105).

The IIT provided a response indicating that workplace influences played a role in translation approaches to produce TIE. In the following statement given as evidence, the IIT twice said, ‘I have to’ and not words such as ‘I chose to’, which may be indicative of institutional bias affecting translation choices:

When they refer to something, but I don’t know the actual meaning or the meaning of that object or that thing, were conceal, then I have to use the word ‘thingy’[…]Ah when—when the meaning—when um—I’m not—I’m unsure about the meaning or the people who are involved in that conversation did not want to reveal what they were talking about, then they will mention a word so I have to choose the word ‘thingy’ (Gilbert, 2014: 108).

Because of IITs not making explicit their rationale for translatory choices, the word ‘thingy’ had become a systemic error appearing (but remaining undetected) in TIE. The mistranslation was not detected due to inadequate transparency of the translation process. This may have resulted in miscarriages of justice that have remained undetected. In addition, research revealed that Chinese IITs working alongside Vietnamese IITs had also adopted the same strategy when producing TIE from the Chinese language, as use of the word ‘thingy’ had become frequent practice within the law enforcement environment, but only in relation to drug-related activity (Gilbert, 2014: 219). Without established standards that assure verifiability, there is a risk that circumstances such as those described above will remain unexposed and will continue to undermine the justice system.

In a recent espionage case in the US, the *translatum* reliability of TIR destined to become TIE at a future trial was challenged by the accused, who was incarcerated pending the commencement of a trial. The accused, a former New York police officer, was released from custody after the prosecution decided not to proceed to trial. The reason cited by the accused person was that the TIR contained unreliable *translatum* (Davis O’Brian, 2023). In this case, the accused was proficient in both Chinese and English and had extensive experience as a law enforcement officer; therefore, it is not surprising that the TIR was subject to significant scrutiny. Importantly, the TIR used to support the Complaint and Affidavit of Arrest Warrant contained only an English translation and did not include a transcript in written Chinese (US District Court Eastern District of New York, 2020: 9). Interestingly, in this case, verifiability was finally achieved, but most likely due to the language skills and background of the accused, and not because of established standards for producing TIE. Accused persons who do not speak the language of the court must rely on others to verify TIR that is intended to be used as TIE against them. Defence teams may have access to government funded legal aid in some jurisdictions to have TIR verified by a suitably qualified translator; however, this is not assured. Therefore, where TIR is not properly verified, the resultant TIE may breach the principle of equality of arms.

The IIT may make inappropriate or incorrect lexical choices during the translation process that may not become transparent unless a translator engaged by the public prosecutor's office, court or the defence identifies a particular bias. For example, the word ‘gear’ is often synonymous with argot of the drug trade. The word ‘gear’ is also synonymous with other English words such as ‘equipment’, ‘things’ or ‘stuff’. In drug-related TIE, however, the translatory choice of the word ‘gear’ instead of other equally or more suitable word choices may affect the reliability of the TIE in terms of the perception instilled in the trier of fact. This was prevalent in an Australian drug-related trial where the IIT was called to give evidence and compelled to give reasons why they chose the word ‘gear’ instead of other suitable word choices. When questioned during a voir dire, the IIT stated that they simply chose the word ‘gear’ without any intention to indicate a drug context (Gilbert, 2014: 107). However, time, money and effort were unnecessarily wasted by the IIT adopting a narrow and definitive approach to the *translatum* process without disclosing areas of potential error or ambiguity.

The examples above demonstrate the necessity and usefulness of translator's notes. Previous research revealed that IITs do not usually declare ambiguity detected in the source language in the form of a translator's comment in the TIE, and the defence may not detect or challenge errors of translation during the trial process. The practice of the IIT independently resolving areas of ambiguity during the translation process without making a translator's comment in the TIE, may mislead the trier of fact resulting in an unfair trial. IIT's comments provide better transparency of the translatory process conducive to the interests of justice, as they establish a basis for verifiability. In Common Law countries, such as Australia and Canada, the voir dire process (Thomson Reuters, 2023) is often used to clarify why the IIT made the choices they did when producing the *translatum*. In Civil Law countries, such as Switzerland and Austria, such clarification can also take place before the court. In addition, in Civil Law countries, there are options to do so much earlier in the criminal proceeding, as prosecutors may play the audio recording during interrogations when confronting the defendant with the evidence. The interpreter and defence lawyer present at the interrogation will have access to the TIR. This proceeding allows prosecutors to provisionally test prospective TIE reliability and allows the defendant to challenge translatory choices made by the IIT.

This paper proposes that efficiencies and reliability can be optimised when the IIT exercises transparency by annotating *translatum* where necessary. Regardless of the source from which the IIT draws extralinguistic context to complete the *translatum* task, any doubt, confusion or apprehension in the mind of the IIT can and should be made transparent through translator's comments. Such a documentary approach makes it easier for the trier of fact to assess the probative value of the evidence. Therefore, TIE reliability is achieved if the product is as loyal as possible to the court and where the IIT provides annotated comments where they assume, paraphrase or contextualise (Nord, 1991: 94s).

## Minimum standards

This paper proposes minimum standards to enhance the quality of IIT contributions to operational requirements and TIE reliability. The authors emphasise that ensuring reliability of TIR before it is presented as TIE is a critical factor that justifies the establishment of minimum standards. In the following presentation, three categories of minimum standards are proposed: general, translatory procedural and (pre-)trial minimum standards.

### General minimum standards

It is important to bear in mind that the skill set for IITs is different from the skill set for court interpreters or for other areas of community interpreting and translating (Capus and Havelka, 2022). NAJIT lists the following additional attributes required of interpreters and translators to effectively undertake the tasks of IIT (NAJIT, 2019):

− acute hearing
− excellent writing skills in both the target and the source language
− good analytic and problem-solving skills
− eye and ear for detail
− good research skills
− a thorough understanding of accepted transcription and translation protocols and practices
− prior experience in transcription and translation
− ability to work well under pressure and meet demanding deadlines
− experience testifying in court as an expert witness
− good working knowledge of pertinent software
− ability to remain neutral and to always adhere to ethical standards.

Unsurprisingly, hearing skills are prioritised. As IITs have little control over the audio quality of the source language, not only must they have the required language skills but they must also have acute hearing skills to discern targeted speech from other voices and extralinguistic sounds. Incorrectly attributing voices to individuals, and consequently attributing statements to the wrong person, is far from being an extraordinary mistake. We propose that IITs should, as in other professions where hearing standards are applied, be required to undertake periodic hearing tests to ensure that they are physically fit to carry out IIT tasks to an acceptable standard.

As for skills such as writing, analysing, researching and protocoling, IITs should be required to successfully complete specifically designed courses ensuring that they satisfy established learning objectives and associated standards prior to being awarded the relevant qualification for ITT. Deficiencies in the training of IITs jeopardise operational effectiveness and undermine the evidentiary process (Capus and Bally, 2020; Gilbert, 2014: 225). Therefore, the authors posit that law enforcement agencies should invest in preparing interpreters and translators for work as IITs by providing courses designed and based on a thorough training needs analysis. Some countries already offer certification training courses that teach basic criminal law, translational and transcribing techniques and other skills.

### Translatory procedural minimum standards

In addition to the above attributes, IITs must also have the professional aptitude and ability to reflect upon and explain their approaches to the translation process. However, it is often the case that IITs are unable to adequately explain the methodology applied to produce the *translatum* (Gilbert, 2014: 112). As a minimum standard, we propose that IITs use the following approach to translation when producing TIR for use as TIE:

1. Make translatory choices by objectively evaluating the meaning potential of the source utterance, taking into consideration the socio-cultural context within which the source utterance takes place.
2. Consider all information available at the time of transcribing and translating.
3. Translate the source text to the best of their ability with the aim of achieving equivalence at word and syntactic levels.
4. Produce the target text to convey cultural components and equivalence of idiomatic language where equivalence of meaning is achievable.
5. Remain faithful to the source language and add translator's comments when they are unsure of intended meaning conveyed in the source language within the context of the language used as the IIT understands it.
6. Provide translator's comments where necessary for the purposes of clarity, verifiability and transparency. Annotations of this nature may include, but are not limited to areas of ambiguity, alternative meanings, alternative lexical and semantic choices, explanation of concepts and idiomatic language that have no equivalent in the target language, and suspected codewords, in addition to reasons for the IIT's choices.

Furthermore, as the working procedure of IITs is closely interconnected with police investigations, it is important to disclose to the trier of fact what kind of instruction is given to IITs in specific cases and contexts. Only upon knowing this input is the applied methodological approach discernible. Such information is important to assess the IIT's input and the impact their contributions have on criminal procedure.

### (Pre-)trial minimum standards

The authors posit that reliability should be examined as early as possible during a criminal proceeding. Law enforcement decisions based on unreliable TIR may lead a violation of a suspect's rights. Also, unreliable TIR containing misleading or incorrect information may result in the ineffective use of resources during an investigation. Therefore, the authors advocate for minimum standards to ensure that errors in TIR are detected as early as possible. Defendants should have access to TIR intended to be used as TIE within a specified minimum timeframe established in law. Also, legal procedures should require an independent IIT or suitably qualified interpreter/translator to review TIR to verify reliability. As charges are brought by the prosecution, we propose that the State should make resources available for the defendant to have an independent translator check the prosecution’s evidence in the form of TIR prior to being admitted as TIE. Alternatively, the State should mandate another IIT, who has not been implicated previously in the TIR production, to conduct quality control of the TIR and verify its reliability prior to TIR being presented as TIE.

The authors emphasise the importance of introducing as a minimum standard that IITs should be permitted, and even obliged, to note and comment whenever they are confronted with terms in the source language that have no direct equivalence in the target language. This would also apply in the case of ambiguous terms, or when the IIT assesses that a term is used as a code for something other than what the word usually represents. IITs should make comment to that effect in the interests of transparency; otherwise, the trier of fact is likely to rely on the TIE in the absence of any challenge raised by the defence.

Having discussed the risks associated with producing TIE and determining translatum reliability, the authors propose the concept of an internationally recognised Translatum Reliability Index (TRI). The Translatum Reliability Index may be designed for useful application by the court, the prosecution, defence and other translators or interpreters who may be engaged in assessing *translatum*. Such an index may include distinct reliability levels depending on the gravity of the defects found. For example, where the acoustic quality of the audio recording is insufficient to clearly identify what has been said by whom (due to inaudibility, extralinguistic noise or interference), the person evaluating the TIR may use a rubrics-based system to allocate a reliability rating on an incremental scale from 1 to 7 or similar. While the general debate as to whether translation is an art or a science (or a combination of both) contributes little to the practical relevance discussed in this paper, a means of scientifically framing the *translatum* within the stringent requirements of evidence law is important. The proposed index may be useful in a move towards quantifying the reliability of a *translatum* aligning with this species of evidence more aptly within the field of forensics.

In conclusion, this study has delved into the critical realm of *translatum* reliability within the context of law enforcement communication surveillance and the criminal justice system. By aligning proposed minimum standards with established principles outlined by the International Association of Prosecutors (IAP, 2023) and the International Association of Defence Counsel (IADC, 2023) this research underscores the paramount importance of safeguarding the rights of the accused and upholding the tenets of a fair trial. The correlation between the reliability of *translatum*, consisting of impartiality and verifiability, and the overall integrity of the justice system has been laid bare, emphasising the potential consequences of distorted meaning and its ripple effect on prosecutorial obligations and the credibility of judicial proceedings.

This paper calls on key stakeholders encompassing police, interpreters, prosecutors, defence lawyers and judges to support the implementation of minimum standards to improve the reliability of intercept evidence. A collective commitment towards rigorous standards and best practice can mitigate the risk of unreliable *translatum* in operational and evidentiary settings. The spotlight is on the need for interdisciplinary collaboration, highlighting the need to distinguish linguistic reliability from evidentiary reliability and contributing to meaningful interdisciplinary research.

Furthermore, the authors acknowledge the pragmatic constraints of time and financial resources, underlining the economic pressures faced by law enforcement authorities. Striking a delicate equilibrium between operational efficiency and the preservation of individual rights, the proposed minimum standards stand as a practical framework for enhancing *translatum* integrity. While the intricacies of language analysis are boundless, this paper underscores the feasibility of the suggested standards to ensure holistic reliability without undue wastage of limited resources.

In the pursuit of justice and truth, the culmination of this research marks a significant stride towards a more transparent and accountable *translatum* framework. By contributing to this evolving discourse, the authors aim to fortify the bedrock of the criminal justice system, ensuring that the words spoken in one language do not distort the pursuit of fairness and truth in another. This paper endeavours to serve as a catalyst for continued exploration, refinement and implementation, ultimately fostering a more just and equitable society.
